# Supplementary material for: What Determines the District-Level Disparities in Immunization Coverage in India: Findings from Five Rounds of the National Family Health Survey
Source: Vaccines (Basel). 2023 Apr 16;11(4):851. doi: 10.3390/vaccines11040851 (PMC10144648; doi:10.3390/vaccines11040851)
Supplement: Supplementary file 1 [file vaccines-11-00851-s001.zip › vaccines-2189305-supplementary.pdf]

Supplementary Materials 1

1. Fairlie Decomposition

The decomposition method proposed by Fairlie is described below. As per the Standard Blinder-Oaxaca decomposition, the higher performing vs. medium performing districts gap in the average value of the dependent variable, Y (here, immunization coverage), can be expressed as

$$\bar{Y}^H - \bar{Y}^M = \left[(\bar{X}^H - \bar{X}^M)\hat{\beta}^H\right] + \left[\bar{X}^M(\hat{\beta}^H - \hat{\beta}^M)\right], \qquad \text{(i)}$$

where, $\bar{X}^j$ Is a row vector of average values of the independent covariates and, $\hat{\beta}^j$ Is a vector of coefficient estimates for immunization coverage j. An extension of this decomposition for a non-linear equation, Y= F $(X\hat{\beta})$, can be written as

$$\bar{Y}^H - \bar{Y}^M = \left[\sum_{i=1}^{N^H} \frac{F(X_i^H\hat{\beta}^H)}{N^H} - \sum_{i=1}^{N^M} \frac{F(X_i^M\hat{\beta}^H)}{N^M}\right] + \left[\sum_{i=1}^{N^M} \frac{F(X_i^M\hat{\beta}^H)}{N^M} - \sum_{i=1}^{N^M} \frac{F(X_i^M\hat{\beta}^M)}{N^M}\right] \qquad \text{(ii)}$$

An equally valid expression for the decomposition is:

$$\bar{Y}^H - \bar{Y}^M = \left[\sum_{i=1}^{N^H} \frac{F(X_i^H\hat{\beta}^M)}{N^H} - \sum_{i=1}^{N^M} \frac{F(X_i^M\hat{\beta}^M)}{N^M}\right] + \left[\sum_{i=1}^{N^H} \frac{F(X_i^H\hat{\beta}^H)}{N^H} - \sum_{i=1}^{N^H} \frac{F(X_i^H\hat{\beta}^M)}{N^H}\right] \qquad \text{(iii)}$$

Where, N^j is the sample size for interest group j. Y^j is the average probability of the binary outcome of the interest group *j*, and *F* is the cumulative distribution function from the logistic distribution. Here, superscripts 'H' and 'M' stand for 'higher performing' and 'medium performing' districts of immunization coverage. Similarly, decomposition analysis was done for "medium-lower" and "higher-lower" performing districts.

In both (ii) and (iii), the first term in brackets represents the part of the gap between districts due to group differences in distributions of the entire set of independent variables, and the second term means the part due to differences in the group processes determining the levels of Y. The second term also captures the portion of the group gap due to districts' differences in unmeasurable or unobserved endowments.

Table S1: **Mean value of the demographic, economic and healthcare utilisation variables by higher, medium and lower performing districts.**

	**Higher performing districts**			**Medium performing districts**			**Lower performing districts**		
		**Confidence Interval (CI)**			**CI**			**CI**	
	Mean	Lower	Upper	Mean	Lower	Upper	Mean	Lower	Upper
**Demographic variables**									
**Sex of child**									
Male	0.52	0.51	0.53	0.52	0.51	0.53	0.50	0.45	0.55
Female	0.48	0.47	0.49	0.48	0.47	0.49	0.50	0.45	0.55
**Birth order**									
1	0.44	0.43	0.44	0.37	0.37	0.38	0.34	0.29	0.38
2	0.36	0.35	0.36	0.33	0.33	0.34	0.36	0.32	0.41
3	0.13	0.13	0.14	0.16	0.16	0.17	0.17	0.13	0.21
4 and above	0.08	0.07	0.08	0.13	0.13	0.14	0.13	0.10	0.17
**Place of residence**									
Urban	0.28	0.28	0.29	0.26	0.26	0.27	0.29	0.25	0.34
Rural	0.72	0.71	0.72	0.74	0.73	0.74	0.71	0.66	0.75
Mother's age at first birth									
19-24	0.31	0.30	0.32	0.33	0.33	0.34	0.33	0.28	0.37
25-34	0.69	0.68	0.69	0.66	0.66	0.67	0.67	0.62	0.71
35-49	0.01	0.00	0.01	0.00	0.00	0.01	0.00	0.00	0.02
**Household Size**									
One-three	0.10	0.09	0.10	0.09	0.09	0.09	0.07	0.05	0.10
Four-five	0.39	0.38	0.40	0.34	0.34	0.35	0.32	0.27	0.36
Six-seven	0.29	0.28	0.29	0.29	0.29	0.30	0.33	0.29	0.38
Eight and above	0.22	0.22	0.23	0.27	0.27	0.28	0.28	0.23	0.32
**Region**									
North	0.18	0.17	0.18	0.10	0.10	0.11	NA	NA	NA
North East	0.01	0.01	0.01	0.05	0.05	0.05	0.25	0.22	0.30
Central	0.16	0.16	0.17	0.35	0.34	0.36	0.29	0.25	0.34
East	0.30	0.29	0.31	0.25	0.24	0.25	NA	NA	NA
South	0.24	0.23	0.24	0.13	0.12	0.13	NA	NA	NA
West	0.11	0.11	0.12	0.12	0.12	0.13	0.45	0.40	0.50
**Socio-economic variables**									
**Mother's education**									
Illiterate	0.14	0.13	0.14	0.22	0.22	0.23	0.15	0.12	0.18
Primary	0.10	0.10	0.11	0.12	0.11	0.12	0.14	0.11	0.18
Secondary	0.56	0.56	0.57	0.50	0.49	0.50	0.61	0.56	0.65
Higher	0.19	0.19	0.20	0.16	0.16	0.17	0.10	0.08	0.13
**Caste**									

ST	0.22	0.21	0.23	0.24	0.24	0.25	0.22	0.18	0.26
SC	0.13	0.12	0.13	0.08	0.08	0.08	0.28	0.24	0.32
OBC	0.41	0.40	0.42	0.45	0.44	0.45	0.24	0.20	0.28
Others	0.24	0.24	0.25	0.23	0.23	0.24	0.26	0.22	0.31
**Religion**									
Hindus	0.79	0.79	0.80	0.80	0.79	0.80	0.62	0.57	0.66
Muslims	0.16	0.16	0.17	0.16	0.15	0.16	0.21	0.17	0.25
Others	0.04	0.04	0.05	0.04	0.04	0.04	0.18	0.14	0.22
**Mother's media exposure**									
No	0.40	0.39	0.41	0.55	0.55	0.56	0.61	0.56	0.65
Partial	0.59	0.58	0.59	0.44	0.43	0.44	0.37	0.32	0.42
Full	0.02	0.01	0.02	0.01	0.01	0.01	0.02	0.01	0.04
**Wealth Quintile**									
Poorest	0.19	0.18	0.20	0.27	0.26	0.28	0.26	0.22	0.30
Poorer	0.20	0.20	0.21	0.22	0.22	0.23	0.24	0.21	0.29
Middle	0.22	0.21	0.23	0.18	0.18	0.19	0.20	0.17	0.25
Richer	0.21	0.21	0.22	0.17	0.17	0.18	0.16	0.13	0.20
Richest	0.18	0.17	0.18	0.15	0.15	0.16	0.13	0.10	0.16
**Healthcare variables**									
**Availability of health card**									
No	0.02	0.02	0.02	0.07	0.06	0.07	0.16	0.13	0.20
Yes	0.98	0.98	0.98	0.93	0.93	0.94	0.84	0.80	0.87
**Distance to health facility problem**									
No	0.77	0.76	0.78	0.74	0.74	0.75	0.73	0.68	0.77
Yes	0.23	0.22	0.24	0.26	0.25	0.26	0.27	0.23	0.32
**Number of antenatal care visit**									
None	0.03	0.02	0.03	0.08	0.08	0.08	0.10	0.07	0.13
One- three	0.24	0.24	0.25	0.42	0.41	0.42	0.38	0.34	0.43
Four and above	0.73	0.72	0.74	0.50	0.50	0.51	0.52	0.47	0.57
**Place of delivery**									
Home	0.05	0.05	0.06	0.12	0.11	0.12	0.11	0.08	0.14
Public facility	0.67	0.66	0.68	0.61	0.60	0.61	0.49	0.45	0.54
Private facility	0.28	0.27	0.29	0.27	0.27	0.28	0.40	0.35	0.44
**Tetanus Toxoid injections**									
No	0.04	0.03	0.04	0.05	0.05	0.05	0.07	0.05	0.10
Yes	0.96	0.96	0.97	0.95	0.95	0.95	0.93	0.90	0.95
**Mother post-natal check**									
Within 2 days	0.15	0.15	0.16	0.16	0.16	0.16	0.15	0.12	0.19
After 2 days	0.85	0.84	0.85	0.84	0.84	0.84	0.85	0.81	0.88
**Baby post-natal care**									
No	0.44	0.43	0.45	0.58	0.58	0.59	0.66	0.61	0.71

Yes	0.56	0.55	0.57	0.42	0.41	0.42	0.34	0.29	0.39
**C-section delivery**									
No	0.71	0.71	0.72	0.80	0.79	0.80	0.85	0.81	0.88
Yes	0.29	0.28	0.29	0.20	0.20	0.21	0.15	0.12	0.19
**Size of child at birth**									
Large	0.19	0.19	0.20	0.19	0.19	0.20	0.23	0.20	0.28
Average	0.70	0.70	0.71	0.69	0.68	0.69	0.61	0.56	0.66
Small	0.10	0.10	0.11	0.12	0.12	0.13	0.15	0.12	0.19
